# Allogenic Adipose Tissue-Derived Stromal/Stem Cells and Vitamin D Supplementation in Patients With Recent-Onset Type 1 Diabetes Mellitus: A 3-Month Follow-Up Pilot Study

**DOI:** 10.3389/fimmu.2020.00993

**Published:** 2020-06-02

**Authors:** Debora B. Araujo, Joana R. Dantas, Karina R. Silva, Débora L. Souto, Maria de Fátima C. Pereira, Jessica P. Moreira, Ronir R. Luiz, Cesar S. Claudio-Da-Silva, Monica A. L. Gabbay, Sergio A. Dib, Carlos E. B. Couri, Angelo Maiolino, Carmen L. K. Rebelatto, Debora R. Daga, Alexandra C. Senegaglia, Paulo R. S. Brofman, Leandra Santos Baptista, José E. P. Oliveira, Lenita Zajdenverg, Melanie Rodacki

**Affiliations:** ^1^Federal University of Rio de Janeiro, Nutrology and Diabetes Department, Federal University of Rio de Janeiro, Rio de Janeiro, Brazil; ^2^Laboratory of Tissue Bioengineering, National Institute of Metrology, Quality and Technology (Inmetro), Rio de Janeiro, Brazil; ^3^Nutrology and Diabetes Department, Federal University of Rio de Janeiro, Rio de Janeiro, Brazil; ^4^Clinical Pathology Service, Federal University of Rio de Janeiro, Rio de Janeiro, Brazil; ^5^Biostatistics Department, Institute of Public Health Studies, Federal University of Rio de Janeiro, Rio de Janeiro, Brazil; ^6^Plastic Surgery Department, Federal University of Rio de Janeiro, Rio de Janeiro, Brazil; ^7^Department of Stem Cell Therapy, Federal University of São Paulo (UNIFESP), São Paulo, Brazil; ^8^University of São Paulo, São Paulo, Brazil; ^9^Hematology Department, Federal University of Rio de Janeiro, Rio de Janeiro, Brazil; ^10^Core Cell Technology, Pontifical Catholic University of Paraná, Curitiba, Brazil; ^11^Surgical Clinic D at University of Sao Paulo, Core Cell Technology, Pontifical Catholic University of Paraná, Curitiba, Brazil; ^12^Multidisciplinary Center for Biological Research (Numpex-Bio), Federal University of Rio de Janeiro, Rio de Janeiro, Brazil

**Keywords:** type 1 diabetes, pancreatic function, transplant, adipose tissue-derived stromal/stem cells, vitamin D

## Abstract

**Objective:** To evaluate the short term safety and potential therapeutic effect of allogenic adipose tissue-derived stromal/stem cells (ASCs) + cholecalciferol in patients with recent-onset T1D.

**Methods:** Prospective, phase II, open trial, pilot study in which patients with recent onset T1D received ASCs (1 × 10^6^ cells/kg) and cholecalciferol 2000 UI/day for 3 months (group 1) and were compared to controls with standard insulin therapy (group 2). Adverse events, C-peptide (CP), insulin dose, HbA1c, time in range (TIR), glucose variability (continuous glucose monitoring) and frequency of CD4^+^FoxP3+ T-cells (flow cytometry) were evaluated at baseline (T0) and after 3 months (T3).

**Results:** 13 patients were included (8: group 1; 5: group 2). Their mean age and disease duration were 26.7 ± 6.1 years and 2.9 ± 1.05 months. Adverse events were transient headache (*n* = 8), mild local reactions (*n* = 7), tachycardia (*n* = 4), abdominal cramps (*n* = 1), thrombophlebitis (*n* = 4), mild floaters (*n* = 2), central retinal vein occlusion (*n* = 1, complete resolution). At T3, group 1 had lower insulin requirement (0.22 ± 0.17 vs. 0.61±0.26IU/Kg; *p* = 0.01) and HbA1c (6.47 ± 0.86 vs. 7.48 ± 0.52%; *p* = 0.03) than group 2. In group 1, 2 patients became insulin free (for 4 and 8 weeks) and all were in honeymoon at T3 (vs. none in group 2; *p* = 0.01). CP variations did not differ between groups (−4.6 ± 29.1% vs. +2.3 ± 59.65%; *p* = 0.83).

**Conclusions:** Allogenic ASCs + cholecalciferol without immunosuppression was associated with stability of CP and unanticipated mild transient adverse events in patients with recent onset T1D.

**ClinicalTrials.gov registration:** NCT03920397.

## Introduction

Type 1 Diabetes (TID) is a chronic disease caused by T-cell mediated autoimmune destruction of pancreatic β-cells. Patients with T1D require lifelong insulin treatment, which may lead to hypoglycemic episodes and interfere in quality of life. Interventions to cure T1D have been pursued. Even preservation or recovery of some residual mass of β cells, albeit not enough to cure the disease, could have potential benefits such as reduction in glycemic variability, severe hypoglycemia, insulin requirement and risk of chronic complications (1–5).

Clinical interventions aiming to cure T1D target the prevention of autoimmune destruction of pancreatic β cells, regression of insulitis, preservation or recovery of β cells residual mass (3, 6, 7). Various therapeutic methods have been evaluated for patients with T1D, including cytostatic drugs, monoclonal antibodies, and pancreas/islets transplantation. They have some limitations in clinical practice, such as adverse events of immunosuppressants, risk of immune rejection and surgical complications (bleeding, portal vein thrombosis and bile leakage) (8–12). Although some of these treatments have initially shown satisfactory outcomes, their effects have not been sustained for long periods and/or their potential side effects limit their repetitive use. In previous studies, most favorable metabolic and pancreatic function outcomes were linked to a better T regulatory cell function (13–15).

Adult stem cells transplantation has emerged as a potential treatment for T1D due to its intrinsic regenerative capacity and immunomodulatory properties. The rationale for its use is to arrest β cells autoimmune destruction and generate functional cells. Mesenchymal stromal/stem cells seem attractive as they have been tested for other autoimmune diseases with promising results (16–24) and do not require immunosuppression, even when allogenic sources are used. *In vivo* and *in vitro* studies showed that MSCs are capable of suppressing immune response by inhibiting the maturation of dendritic cells, suppressing T cells function and inducing expansion of regulatory T cells (16–19). A recent meta-analysis of the clinical efficacy and safety of stem cell therapy for T1D indicated that the treatment seems relatively safe and effective, but most studies are small, use hematopoietic stem cells with immunosuppression and autologous origin (20). In that meta-analysis, patients with recent-onset T1D that received MSCs (from bone marrow or umbilical cord tissue) did not have significant reduction in HbA1c or improvement in C-peptide levels, but 20% of treated T1D patients achieved exogenous insulin independence at some point (20). Adipose tissue-derived stromal/stem cells (ASCs) have not been evaluated for this purpose.

ASCs are an abundant source of adult stromal/stem cells, easily accessible by liposuction. These cells seem to display more potential immunosuppressive properties than other mesenchymal stem cells, with more pronounced cytokines secretion, suggesting a promising therapeutic application in autoimmune diseases, such as T1D. As ASCs do not express co-stimulatory molecules on their surface, they are unable to activate alloreactive T cells and could therefore be used for allogenic transplantation without the need for immunosuppression (18, 19). Studies that tested ASC for musculoskeletal disorders, perianal fistula in Crohn's disease and psoriasis showed potential therapeutic effects (21–23). Their use is currently been tested for autoimmune diseases, especially multiple sclerosis (24, 25).

Vitamin D (VitD) seems to have immunomodulatory effects. *In vitro* and *in vivo* studies showed that sufficient levels of VitD could preserve residual β cells and insulin secretion. VitD appears to inhibit lymphocyte proliferation, inhibit cellular autoimmune pathways and stimulate T regulatory response (26–28). However, results with the use of vitamin D for patients with T1D are still inconsistent (29–31).

Since T1D pathogenesis is multifactorial, interventions to approach islet autoimmunity should probably include a combination of agents with different mechanisms of action. Some authors have already suggested that acting at different points of the autoimmune process is more effective than treatment with a single therapy (32–34). The agents used for intervention in patients with T1D should have the lowest possible toxicity potential, especially if periodic repetition of the proposed treatment is considered. Our aim was to evaluate the short-term safety and efficacy of ASCs infusion from healthy donors and daily cholecalciferol (VitD) supplementation in patients with recent-onset T1D, a combined therapy that offers the opportunity of immunomodulation without the need of immunosuppression.

## Research Design and Methods

### Patients and Study Design

This is a prospective, single-center, open trial, phase II, in which patients (Group 1) with recent onset T1D received a single dose of allogenic adipose tissue derived stem/stromal cells (ASCs) and cholecalciferol (Vit D) 2,000 IU/day for 3 months or were included in a control group that received standard insulin therapy with multiple injections (Group 2). This trial was registered at ClinicalTrial.gov (NCT03920397) and approved by Ethics Research Board of the University Hospital Clementino Fraga Filho (HUCFF)/ Federal University of Rio de Janeiro (UFRJ). Participants or their legal representatives (in minors) signed an informed consent before inclusion (protocol 17488313.1.0000.5257).

Inclusion criteria were: diagnosis of T1D according to American Diabetes Association (ADA) criteria for < 4 months; age between 16 and 35 years; pancreatic autoimmunity (anti glutamic acid decarboxylase or GADA+). Exclusion criteria were: current or prior malignant diseases; pregnancy or desire to become pregnant within 12 months of the study; breastfeeding; HIV(+), Hepatitis B or C (+); diabetic ketoacidosis at diagnosis; glomerular filtration rate < 60 ml/min and use of immunosuppressors or glucocorticoids. Similarly to Voltarelli JC and Couri CE, we decided to exclude patients with previous diabetic ketoacidosis as the first patient included in their trial with diabetic ketoacidosis failed to benefit from AHST (35).

### Stem/Stromal Cells Differentiation Procedures

ASCs differentiation potential into adipocytes, osteoblasts, and chondrocytes, were evaluated in third passage with a commercial medium (Lonza, Walkersville, EUA). The medium inducer was changed every 3 days during 3 weeks. To adipogenic and osteogenic differentiation, cells were seeded on glass coverslips (Sarstedt, Newton, NC, USA) in 24-well plates (Sarsted). Briefly, cells were treated with Bouin's fixative (Biotec, Labmaster, Paraná, Brazil) for 10 min, washed twice with 70% ethanol and once with Milliq water. Oil Red O (Sigma-Aldrich) was used to visualize lipid-rich vacuoles and hematoxylin-eosin (HE) (Biotec) was used for nuclear staining. Osteogenic differentiation was evaluated by Alizarin Red S (Fluka Chemie, Buchs, UK) and light green (Sigma-Aldrich) was used to counterstain. For chondrogenic differentiation, cells were grown in micromass culture. Briefly, 5 × 105 cells in 0.5 ml of medium were centrifuged at 300 g for 10 min in a 15 mL polypropylene tube to form a pellet. Without disturbing the pellet, cells were cultured for 21 days with medium inducer. On day 21, cell aggregates were fixed in 10% formaldehyde for 1 h dehydrated in serial ethanol dilutions and embedded in paraffin blocks. Toluidine Blue staining (Sigma-Aldrich) demonstrate the presence of intracellular matrix proteoglycans. Control cells were kept in DMEM-F12 medium with 15% FCS.

### Stem/Stromal Cells Lipoaspirate and Culture

Adipose tissue samples were obtained through liposuction procedures of three healthy female individuals undergoing aesthetic surgery at *Hospital Universitário da Universidade Federal do Rio de Janeiro*, RJ, Brazil. Donors'sorology was negative for syphilis, Chagas disease, Hepatitis B virus (HBV), Hepatitis C, HIV 1 and 2, HTLV I/II. All donors were Cytomegalovirus (CMV) IgG positive with PCR negative in blood samples and in ASCs.

ASCs were isolated, cultured and characterized as previously described (18). Samples were processed at Core Cell Technology of Pontifícia Universidade Católica do Paraná. Briefly, 100 ml of adipose tissue were washed in sterile phosphate-buffered saline (PBS) (Gibco Invitrogen). One-step digestion by 1 mg/ml collagenase type I (Invitrogen) was performed for 30 min at 37°C during permanent shaking, followed by filtration through 100 μm mesh filter (BD FALCON, BD Biosciences Discovery Labware, Bedford, MA, USA). Cell suspension was centrifuged at 800 g for 10 min, and erythrocytes were removed by lysis buffer, pH 7.3. The remaining cells were washed at 400 *g* for 10 min and cultured at a density of 1 × 10^5^ cells/cm^2^ in T75 culture flasks and DMEM-F12 (Gibco Invitrogen) supplemented with 10% of fetal calf serum (FCS), penicillin (100 units/ml) and streptomycin (100 μg/ml). Culture medium was replaced 3 days after seeding, and then twice a week. ASCs were sub-cultured after reaching 80% confluence, with 0.5% trypsin/EDTA (Invitrogen) solution. Cells were replated at a density of 4 × 10^3^ cells/cm^2^ for expansion.

Quality control of cell suspension sterility was evaluated by tests to detect bacteria and fungi (Bact/Alert 3D, Biomerieux), endotoxins (Endosafe™ PTS, Charles River) and Mycoplasma (KIT MycoAlert™ PLUS Mycoplasma Detection, Lonza). Cell viability was performed by flow cytometry using vital dye 7-AAD (7-Aminoactinomycin D—BD#559925) to determine percentage of viable cells and Annexin V protein (BD#51-65875X) to determine percentage of cells in apoptosis. Cytogenetic analysis was performed by GTG-banding method.

Cells were characterized by flow cytometry before the clinical application, using the following monoclonal antibodies: FITC-labeled CDç/14 (BD#555397), CD45 (BD#555482), CD19 (BD#555412), CD44 (BD#555478); PE-labeled CD73 (BD#550257), CD90 (BD#555596), CD166 (BD#559263), PerCP-labeled HLA-DR (BD#551375); APC-labeled CD34 (BD#555824), CD105 (BD#562408), CD29 (BD#559883) (BD Pharmingen). At least 100.000 events were acquired on BD FACSCalibur™ flow cytometer (BD Biosciences), and data were analyzed using FlowJo 10 (TreeStar) software^11A^ (Supplementary Material 1).

### ASCs Infusion

At the day of infusion, ASCs monolayer were dissociated as described above and 1 × 10^6^ cells/kg of the donor patient were suspended in 5 ml of saline solution with 50% albumin and 5% ACD (Anticoagulant Citrate Dextrose Solution). Cell suspension was sent to the hospital in cooler with recycled ice.

An aliquot of cells was evaluated after transportation for monitoring viability and phenotype of ASCs by flow cytometry. Cells were washed with PBS-BSA 3% (bovine serum albumin) and incubated at 4°C for 30 min with the following monoclonal antibodies conjugated to fluorescent dyes: CD105-FITC (fluorescein isothiocyanate), CD73-PE (phycoerythrin) and CD90-APC (allophycocyanin), all from BD Biosciences, Franklin Lakes, NJ, USA. Unstained cells were used as controls. Then, cells were washed with PBS-BSA 3% and incubated with 7AAD (7 Amino Actinomycin D). Twenty thousand events were acquired in FACSAria III cytometer (BD Biosciences) and data were analyzed using FACSDiva 8.0 software (BD Biosciences). The percentage of viable cells was estimated by 7AAD exclusion.

Patients that received ASCs were admitted into hospital in the day of the infusion. A single dose of ASCs was infused in a peripheral upper arm vein during 15–20 min. Patients were discharged from hospital 24 h after infusion. Patients started taking oral cholecalciferol 2,000 IU in the same day.

### Clinical and Biochemical Evaluation

All participants were followed for 3 months. At baseline, they were interviewed and had a physical exam. Weight, height, body mass index (BMI), blood pressure, heart frequency, frequency of hypoglycemia and insulin dose/kg of body weight were evaluated in the first visit (T0) and after 1 (T1) and 3 (T3) months. Insulin dose adjustments were done at each visit as necessary, according to glycemic control. Cases and controls received the same diabetes education, nutritional recommendations and help with management from health care providers. Honeymoon phase was defined as insulin dose ≤ 0.5 IU/Kg and HbA1C < 7.5% (36). Blood samples were collected prior to ASCs infusion and at T1 and T3 for the following measurements: HbA1c—Glycated hemoglobin method- HPLC- High Performance Liquid Chromatography by boronate affinity (Trinity Biotech, USA), blood count and biochemistry analysis. Pancreatic function was evaluated through C-Peptide (CP) measurement (Microparticle Chemiluminescent Immunoassay method, Architect Abbott, Spain, following the manufacturer's protocol) 0 (basal; 8 h fasting), 30, 60, 90, and 120 min after a liquid mixed meal (Glucerna®). The area under the curve (AUC) was calculated. Insulin usual dose was administered after each test was completed. All tests were performed with fasting glucose between 70 and 250 mg/dL. Glutamic acid decarboxylase antibodies (GADA) and Islet Tyrosine Phosphatase 2 (IA2) were analyzed (ELISA-human antibodies tested by quantitative ELISA method—Enzyme-Linked Immunosorbent Assay, Euroimmun brand and Molecular Devices Spectra max reader, Germany).

### Glucose Variability Assessment

Retrospective analysis of 72 h continuous glucose monitoring system (CGM—*Ipro Medtronic*) data was performed in 7 T1D patients and 4 controls at T0 and T3.

Mean glucose, glucose standard deviation (SD), J-Index, M-value, glycemic risk assessment in diabetes equation (GRADE), high blood glucose index (HBGI), low blood glucose index (LBGI), mean amplitude of glucose excursions (MAGE), mean of daily differences (MODD) and continuous overall net glycemic action at 1 h (CONGA 1) were calculated as described in original publications (37–39). The percentage of glucose reading in time in range (TIR) established as 70–180 mg/dL was recorded (40).

All volunteers received individual face-to-face consultation sessions, which included individualized diet prescriptions based on *American Diabetes Association* current recommendations (dietary energy content of 45–55% carbohydrates, 15–20% protein, and 25–35% total fat, ≤7% saturated fatty acids (SFA), 5–15% monounsaturated fatty acids (MUFA); ≤10% polyunsaturated fatty acids (PUFA), and 30–50% total fiber intake) (41) and advice on food selection and carbohydrate counting method. During CGM period, all foods and drinks consumed were documented.

### Flow Cytometry

Mononuclear cells were isolated from peripheral blood samples by density centrifugation on Ficoll (Ficoll-Paque, GE Healthcare). Blood samples diluted in an equal volume of phosphate buffered saline (PBS) 0.01 M were overlaid on Ficoll-Paque and centrifuged at 500 g for 15 min, with centrifugation stop break turned off. The ring of mononuclear cells formed in tubes was harvested, washed twice with PBS containing 3% of bovine serum albumin (3% PBS-BSA) and incubated for 30 min at 4°C, shielded from light, with the following fluorochrome-conjugated anti-human monoclonal antibodies: CD45RA-PE (Clone HI100), CD3 PE-CF594 (Clone UCHT1), CD4-PERCP CY5.5 (Clone RPA-T4) and CD8-APCH7 (Clone SK1), with anti-human FoxP3—Alexa 647 (Clone 259 D/C7) (BD Biosciences, Franklin Lakes, NJ, USA). Cells were washed with PBS and followed the intracellular FoxP3 staining protocol, according to manufacturer's recommendations (BD Biosciences). Briefly, surface-stained cells were fixed and permeabilized using the buffers from Human FoxP3 Buffer Set (BD Biosciences). Cells were washed in PBS and incubated with anti-human FoxP3-Alexa 647 antibody (BD Biosciences) for 30 min at room temperature in the dark. After the incubation period, cells were washed with PBS and treated with 0.5 mL of BD FACS™ Lysing Solution (BD, Biosciences) during 10 min at room temperature, to remove residual erythrocytes. A hundred thousand events were acquired on FACSAria III (BD Biosciences), which was calibrated using Cytometer Settings and Tracking (CST) beads (BD Biosciences) according to the cytometer manufacturer's recommendations. Background staining was determined using unstained cells. An FMO (Fluorescence Minus One) control was used to set the boundary between negative and positive fluorescence for FoxP3. Cells incubated with a single antibody coupled with a fluorescent dye were used for compensation purposes. Data were analyzed using FACSDiva 6.0 software. A gate on lymphocytes was defined in a forward scatter (FSC) vs. side scatter (SSC) dot plot, followed by gating on CD45RA^+^CD3^+^ lymphocytes, followed by gating on CD4^+^ or CD8^+^ cells. Finally, a gate was set to determine the percentage of FoxP3^+^ cells among CD45^+^CD3^+^CD4^+^ cells or CD45^+^CD3^+^CD8^+^ cells.

T-cells were evaluated on blood samples before (T0), one (T1) and three (T3) months after ASCs infusion.

### Safety Tests

Adverse events were recorded during the procedure and during the hospitalization. At each outpatient visit (T0, T1, and T3), patients underwent an interview, clinical exam and biochemical evaluation to monitor for adverse events. Laboratorial evaluation included: blood count, lipids, renal, and hepatic function, TSH, free tyroxine, calcium, phosphorus and 25 OH vitamin D (performed using the automated biochemical equipment CMD 800 IX1).

### Statistical Analysis

The primary outcome was the presence of severe adverse events. The secondary outcomes were other adverse events, changes in insulin uses, A1c and CP AUC. The sample size was established by convenience sampling according to the number of cells that were available for the intervention procedure. Data are expressed as mean +/– standard deviation. Descriptive statistics have been used to summarize patient's characteristics. Comparisons of categorical variables have been performed by means of Chi Square test. Continuous variables have been compared by Mann-Whitney test. Wilcoxon test were used to compare results at baseline and after follow-up. Statistical tests are based on a 2-sided significance level of 0.05. SPSS software, version 21.0 was used for statistical analyses.

## Results

### Characteristics of the Study Group

**Figure 1:**
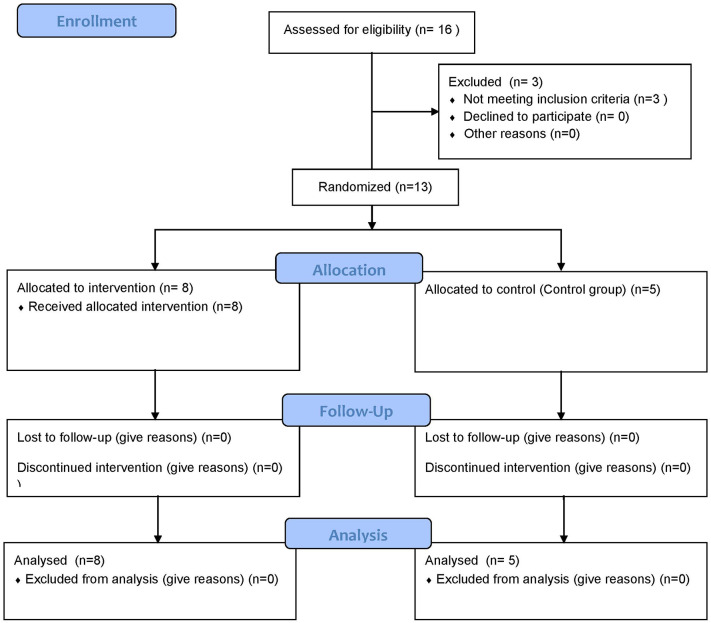
Flow Diagram of the clinical trial.

Sixteen newly diagnosed T1D volunteers fulfilled inclusion criteria and agreed to participate. Three were excluded due to glucocorticoid use, impaired renal function and pulmonary tuberculosis. Thirteen cases were evaluated: eight patients received ASCs infusion + vitamin D (Group 1) and five were included as controls (Group 2) (Figure 1). The study was disclosed in brazilian endocrinologists social media. The first five candidates were included in group 1. The next eight were included by randomization. All completed 3 months of follow-up.

Clinical characteristics of the study group are described in Tables 1, 2. The mean age was 26.75 ± 6.11 years for group 1 and 20.6 ± 3.84 years for group 2 (*p* = 0.04). Their disease duration were 2.9 +/−1.05 and 3.0 ± 0.70 months, respectively (*p* = 0.84). All received insulin through subcutaneous injections. Biochemical, metabolic and immunological characteristics before and after intervention are reported in Table 2.

**Table 1:** Characteristics of the study group.

**Group**	**Age (Gender)**	**Ethnicity**	**BMI**	**T1D**	**ASCs**	**Ins**	**Ins**	**Ins**	**HbA1C**	**HbA1C**	**HbA1C**	**CP**	**CP**	**CP**
						**T0**	**T1**	**T3**	**T0**	**T1**	**T3**	**T0**	**T1**	**T3**
#1 (G1)	26 (M)	W	26.06	4	78	0.84	0.64	0.47	9.90	7.60	6.20	104.85	63.60	157.05
#2 (G1)	35 (M)	NW	25.91	4	74	0.17	0.15	0.18	8.0	7.50	6.60	148.95	162.60	149.70
#3 (G1)	28 (M)	W	23.38	2	65	0.21	0.18	0.17	6.30	5.60	5.50	340.50	470.7	388.95
#4 (G1)	34 (F)	W	23.56	2	73	0.07	0.07	0.11	7.50	6.90	6.20	328.50	317.7	291.90
#5 (G1)	23 (F)	NW	20.76	1.7	60	0.15	0.15	0.12	7.90	6.30	6.40	178.95	158.85	99.30
#6 (G1)	16 (F)	W	20.96	3.5	55	0.47	0.47	0.49	6.90	5.80	5.70	318.75	354.30	251.10
#7 (G1)	28 (F)	W	23.71	2	69	0.25	0.20	0.25	7.60	6.60	6.90	233.70	270.00	243.45
#8 (G1)	24 (M)	W	26.06	4	66	0.30	0.18	0	7.40	7.20	8.30	153.00	204.00	108.15
#9 (G2)	16 (M)	W	18.25	2	–	0.92	0.82	0.92	10.6	6.90	7.80	86.25	140.70	173.70
#10 (G2)	20 (F)	NW	23.71	3	–	0.92	0.69	0.64	6.80	6.80	6.90	90.30	127.95	78.75
#11 (G2)	18 (M)	NW	18.20	3	–	0.60	0.60	0.70	6.90	7.40	7.20	102.75	118.65	106.20
#12 (G2)	25 (F)	W	20.60	3	–	0.20	0.20	0.20	7.70	7.10	8.20	160.2	147.90	123.15
#13 (G2)	24 (M)	W	19.30	3	–	0.50	0.50	0.60	10.10	7.40	7.30	113.25	101.55	48.45

*Body mass index (BMI) expressed as kilogram of body weight/centimeter^2^ height was measured at the time of the transplant. ASCs number of cells was calculated according to patient weight × 10^6^. G1, patient that received ASCs + VitD or Group 1; G2, control or Group 2; W, white; NW, non-white; T1D, Type 1 Diabetes duration in months; T0, basal period, T1, 1 month after intervention, T3, 3 months after intervention; Ins, insulin dose (IU/Kg); A1C, glycated hemoglobin (mg/dl,%); CP, the area under the curve of C-Peptide (ng/ml)*.

**Table 2:** Comparison between group 1 and 2 before and after ASCs infusion + VitD.

**Indexes**	**T0**			**T1**			**T3**		
	**Group 1**	**Group 2**	***P*-value**	**Group 1**	**Group 2**	***P*-value**	**Group 1**	**Group 2**	***P*-value**
HbA1C (mg/dl,%)	7.68 ± 1.04	8.42 ± 1.80	0.36	6.68 ± 0.75	7.12 ± 0.27	0.24	6.47 ± 0.86	7.48 ± 0.52	**0.03**
Insulin dose (IU/Kg)	0.31 ± 0.24	0.62 ± 0.30	0.08	0.25 ± 0.19	0.56 ± 0.23	**0.02**	0.22 ± 0.17	0.61 ± 0.26	**0.01**
GADA (mIU/L)	261.69 ± 138.77	182.13 ± 130.06	0.32	674.33 ± 1348.57	144.87 ± 151.81	0.41	697.75 ± 1338.72	245.08 ± 219.77	0.47
IA2 (mIU/L)	158.28 ± 270.59	818.74 ± 1718.42	0.31	156.68 ± 267.33	817.66 ± 1779.01	0.31	163.19 ± 280.34	815.77 ± 1780.05	0.32
TSH (mIU/L)	3.51 ± 3.06	3.76 ± 2.47	0.88	3.34 ± 3.30	3.48 ± 2.32	0.94	3.27 ± 3.08	3.45 ± 2.57	0.92
Vitamin D (ng/ml)	35.31 ± 14.07	21.74 ± 7.03	0.07	39.42 ± 6.28	25.35 ± 7.22	**0.01**	46.96 ± 8.13	24.73 ± 4.80	**0.01**
CD4+ FOXP3+ (%)	10.22 ± 7.14	15.28 ± 8.34	0.27	17.55 ± 6.29	11.72 ± 3.18	0.12	14.62 ± 8.97	10.76 ± 6.27	0.42
CD8+ FOXP3+ (%)	13.85 ± 9.74	5.52 ± 2.78	0.09	15.85 ± 7.33	5.87 ± 1.85	**0.03**	10.72 ± 9.04	11.25 ± 9.02	0.09
CP AUC (ng/ml min)	225.90 ± 92.91	110.55 ± 29.72	**0.02**	250.22 ± 129.49	127.35 ± 18.31	**0.03**	211.20 ± 100.42	106.05 ± 47.25	0.06
Media	6.94 ± 1.44	7.37 ± 1.13	0.66	−−	−−	–	6.79 ± 1.87	6.94 ± 0	0.94
SD	1.66 ± 0.69	1.84 ± 1.05	0.75	−−	−−	–	1.67 ± 0.93	2.36 ± 0	0.51
M-Value	0.95 ± 5.02	3.61 ± 2.96	0.42	−−	−−	–	1.02 ± 5.48	9.19 ± 0	0.20
MAGE	1.31 ± 0.34	0.59 ± 0.27	0.56	−−	−−	–	1.14 ± 0.80	0.67 ± 0.28	0.95
J Index	25.58 ± 13.15	29.39 ± 11.42	0.67	−−	−−	–	25.53 ± 16.88	29.00 ± 0	0.85
MODD	−−	−−	–	−−	−−−	–	2.20 ± 0.68	3.27 ± 0	0.50
GRADE	0.61 ± 0.82	0.61 ± 0.32	0.99	−−−	−−	–	256.69 ± 722.05	0.99 ± 0	0.75
LBGI	2.75 ± 1.97	2.77 ± 2.20	0.99	−−	−−	–	2.57 ± 1.87	6.17 ± 0	0.11
HBGI	4.55 ± 4.38	3.87 ± 2.75	0.81	−−	−−	–	3.92 ± 4.23	5.82 ± 0	0.68
CONGA-1	1.36 ± 1.02	1.91 ± 0.47	0.41	−−	−−	–	1.37 ± 0.79	1.83 ± 0	0.60
Time in range (%)	92.71 ± 4.27	81.00 ± 18.16	0.12	−−	−−	–	89.43 ± 13.50	57.50 ± 9.19	0.11

*Glycemic variability indexes are expressed in mmol/L. Data are expressed as mean ± standard deviation. T0, basal period, T1, 1 month after intervention, T3, 3 months after intervention, HbA1C, glycated hemoglobin; GADA, Glutamic acid decarboxylase antibodies; IA2, Islet Tyrosine Phosphatase 2; CP AUC, the area under the curve of C-Peptide; SD, standard deviation; MAGE, mean amplitude of glucose excursions; MODD, mean of daily differences; GRADE, glycemic risk assessment in diabetes equation; LBGI, low blood glucose index; HGBI, high blood glucose index; CONGA-1, continuous overall net glycemic action at 1 h. GADA and IA2A titers were considered positive > 10I U/ml. Bold values means p value ≤ 0.05*.

### ASCs Infusion

**Figure 2:**
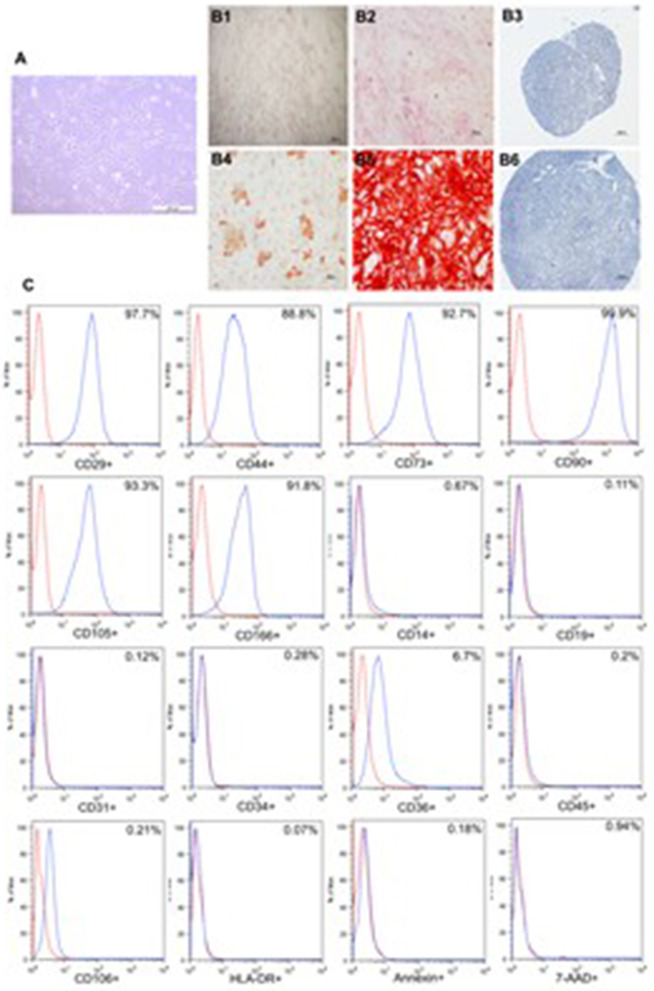
Characterization of adipose tissue-derived stromal/stem cells. **(A)** Adipose tissue-derived stromal/stem cells morphology. **(B)** BM-MSC differentiation. Cells were incubated for 21 days in the presence of specific differentiation agents for adipocytes, osteoblasts and chondrocytes. Differentiation into adipocyte lineage was demonstrated by staining with Oil Red O **(B4)**, Alizarin Red S staining shows mineralization of the extracellular matrix in osteogenic differentiation **(B5)** and toluidine blue shows the deposition of proteoglycans and lacunaes in chondrogenic differentiation **(B6)**. Untreated control cultures without adipogenic, osteogenic or chondrogenic differentiation stimuli are shown **(B1–B3)**. **(C)** Representative figure of the flow cytometric analysis. The red line indicates isotype control and the blue line represent.

Differentiation to adipocytes, osteoblasts, and chondrocytes was qualitatively assessed based on cell morphology and cytochemistry (Figure 2).

After 21 days, large, rounded cells with cytoplasmic lipid-rich vacuoles, for adipogenic differentiation were observed. Osteogenic differentiation was assessed by mineralization of extracellular matrix. In chondrogenic differentiation assays, the presence of intracellular matrix proteoglycans and chondrocyte-like lacunae were observed. Untreated control cultures did not exhibit spontaneous differentiation after 21 days of cultivation.

Group 1 (*n* = 8) received intravenous transfusion of ASCs (mean dose: 67.37± 7.65 × 10^6^ cells). ASCs were characterized by immunophenotyping, and mean percentage labeling of cells was as follows: CD105 93.47%; CD73 96.20%; CD90 99.77%; CD29 98.83%; CD166 94.75%; CD44 89.76%; CD14 1.57%; CD34 0.52%; CD45 0.84%; CD19 0.64% and HLA-DR 0.68%. All tests for microorganism's growth control were negative. All samples were approved by cytogenetic quality control for therapeutic use and no clonal chromosomal rearrangements were detected. After isolation and culture, 95.1% of viability was obtained.

### Adverse Events

No serious adverse events were observed. Patients in group 1 presented the following immediate transient adverse events: transient headache (*n* = 8), mild local infusion reactions (*n* = 7), tachycardia (*n* = 4), and abdominal cramps (*n* = 1). Four patients developed local superficial thrombophlebitis within the first week without systemic manifestations and two reported transient mild eye floaters during infusion, with no subsequent visual abnormalities. One patient developed central retinal vein occlusion at T3, with complete resolution.

### Pancreatic Function

**Figure 3:**
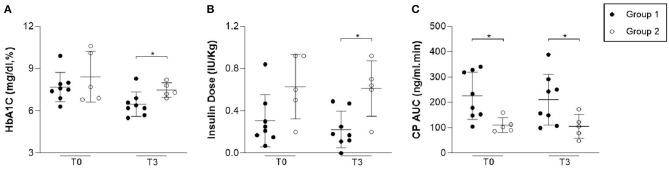
Levels of HbA1C, insulin dose and C-Peptide during follow-up. The levels were evaluated on blood samples before (T0) and three (T3) months after ASCs infusion. Group 1 presented lower HbA1C (*p* = 0.03) and insulin dose (*p* = 0.01) in comparison to group 2. Group 1 presented similar CP AUC (*p* = 0.06) in comparison to group 2.

At baseline, group 1 presented higher CP levels than group 2 (225.90 ± 92.91 vs. 110.55 ± 29.72 ng/ml; *p* = 0.02). At T1, the difference between groups was significant (250.22 ± 129.49 vs. 127.35 ± 18.31 ng/ml; *p* = 0.03), which was not observed at T3 (211.20 ± 100.42 vs. 106.05 ± 47.25 ng/ml; *p* = 0.06) (Table 2 and Figure 3). There was a correlation between CP at T0 and at T3 (*p* = 0.03; *R* = 0.66). Groups 1 and 2 had similar percentage of CP AUC decrease during follow-up (−4.6 ± 29.1% vs. +2.3 ± 59.65%; *p* = 0.83). Neither cases or controls had significant changes in CP over time (*p* = 0.57 e *p* = 0.68).

### Glycemic Control

At baseline, there were no significant differences in HbA1c (7.68 ± 1.04 vs. 8.42 ± 1.8%; *p* = 0.60), insulin dose (0.31 ± 0.24 vs. 0.62 ± 0.30 IU/Kg; *p* = 0.06) or TIR (92.71 ± 4.27% vs. 89.42 ± 13.5%; *p* = 0.35) between groups. One month after infusion, 2 patients in group 1 became insulin free, for 4 and 8 weeks each. At this time, lower insulin requirement were observed in group 1 when compared to group 2 (T1: 0.25 ± 0.19 vs. 0.56 ± 0.23 IU/Kg, *p* = 0.02) and this difference persisted after 3 months (T3: 0.22 ± 0.17 vs. 0.61 ± 0.26IU/Kg, *p* = 0.01). At T3, group 1 also had lower HbA1c than group 2 (6.47 ± 0.86 vs. 7.48 ± 0.52%; *p* = 0.03). Mean glucose readings in TIR were 89.43 ± 13.5 in group 1 vs. 57.50 ± 9.19% in group 2 (*p* = 0.11). The decline in HbA1c over time was significant in cases (*p* = 0.04) but not in controls (*P* = 0.68) and changes in insulin dose were not significant in either of the groups (*p* = 0.27 and *p* = 1.00). Moreover, at T3 all patients (*n* = 8) of group 1 were in honeymoon phase vs. none in group 2 (*p* = 0.01). Both groups had similar percentage of HbA1C reduction (−14.79 ± 13.68% vs. −8.37 ± 17.1%; *p* = 0.72) without statistically significant differences in insulin requirement (−14.48 ± 13.68% vs. +1.2 ± 19.97%; *p* = 0.52). Table 2 and Figure 3 summarizes these results. Neither CP at T0 or age were correlated to the rate of decline in HbA1c (*p* = 0.57; *R* = 0.17 and *p* = 0.87; *R* =−0.04). There was an inverse correlation between insulin dose requirement and Vit D levels at T1 (*p* = 0.05; *r* = 0.58) and T3 (*p* = 0.01; *r* = 0.77). Although there was no association between vitamin D and HbA1c at T1 (*p* = 0.99; *r* = 0.01), a negative correlation was found at T3 (*p* = 0.03; *r* = 0.67).

Retrospective analysis of blinded 72 h CGM did not show significant differences in SD, M-Value, MAGE, J-Index, MODD, GRADE, LBGI, HBGI, CONGA-1 between groups, as shown in Table 2. Glucose profiles are shown in Supplementary Material 2.

### Frequency of FOXP3-Expressing Lymphocytes

**Figure 4:**
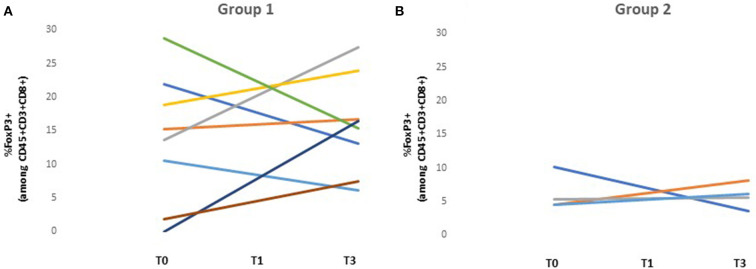
Variation of FoxP3+ cells among CD45+CD3+CD8+ cells. The levels of T-cells were evaluated on blood samples before (T0), one (T1) and three (T3) months after ASCs infusion. **(A)** Individual variation of FoxP3+ cells among CD45+CD3+CD8+ cells of group 1. **(B)** Individual variation of FoxP3+ cells among CD45+CD3+CD8+ cells of group 2. At T1, group 1 presented a higher frequency of CD45+CD3+CD8+FoxP3+ cells in comparison to group 2 (*p* = 0.03). At T0 and T3 no significant difference was found (*p* = 0.17 and *p* = 1.00, respectively).

There was no significant difference between groups for % CD4+FoxP3^+^cells, but group 1 showed higher frequency of CD8+FoxP3^**+**^ cells at T1 when compared to group 2 (*p* = 0.03), as shown in Table 2 and Figure 4.

There was a negative correlation between %CD4+FoxP3+ cells and: (1) HbA1C (*p* = 0.01; *r* = 0.71), (2) mean glucose (*p* = 0.02, *r* = 0.77); (3) SD (*p* = 0.01, *r* = 0.82); (4) M-Value (*p* = 0.02, *r* = 0.03); (5) J-Index (*p* = 0.01, *r* = 0.02); (6) HBGI (*p* = 0.01, *r* = 0.87) and (7) MODD (*p* = 0.04, *r* = 0.77) at T3. No significant correlation was found between % CD4+FoxP3+ cells and MAGE (*p* = 0.77; *r* = 0.12). Frequency of CD4+FoxP3+ cells was positively correlated to TIR (*p* = 0.04; *r* = −0.76).

At T1, %CD8+FoxP3+ cells were positively correlated with CP (*p* = 0.04; *r* = 0.72). At T3, a negative correlation was found between % CD8+FoxP3+ vs. HbA1C (*p* = 0.04; *r* = 0.73) and HBGI (*p* = 0.02, *r* = 0.78).

Additional data are shown in Supplementary Materials 1, 2.

## Discussion

We evaluated the short-term safety of ASCs infusion from healthy donors and daily oral cholecalciferol in patients with recent-onset T1D, as well as their potential therapeutic effect on glycemic control and pancreatic function. In most cases, mild and transient adverse events were observed (local reactions, tachycardia), except for one patient that presented central vein occlusion 3 months after infusion, with complete resolution. This reaction was probably not associated with the therapy since it occurred months after the intervention. A higher risk for this condition has been previously reported in individuals with diabetes (42) but the association between recent-onset T1D and this condition has not been shown. Two cases of central retinal vein occlusion have been previously reported in patients with hematologic malignancies receiving hematopoietic stem cell transplantation but not with adipose tissue-derived stromal/stem cells (43, 44). Tachycardia and thrombophlebitis may be associated with high cellularity concentration, high viscosity or other cell stabilizing products.

This was the first trial to test allogenic ASCs without immunosuppression plus VitD supplementation in newly diagnosed T1D patients. Allogenic source of cells was chosen due to the possibility of impairment of mesenchymal stromal/stem cells immune properties in individuals with T1D (44–46). The use of allogenic cells in this short-term evaluation was associated with mild and transient unanticipated adverse events, although a longer follow-up is still required.

After a 3 month follow-up, those that underwent ASCs infusion + VitD had a better glycemic control and lower insulin requirement than the group in standard treatment. We cannot exclude that the better glycemic control in the treated individuals are due to the higher baseline CP or slightly older age, which have been associated with more sustained residual β-function in previous studies (5, 47). However, neither basal CP or age were correlated to A1c decrease in this study and the sample comprised mostly young adults. Moreover, separate longitudinal analyses were performed for each group and indicated an improvement in HbA1c in the intervention group but not in controls. This suggests that the intervention itself might have a potential therapeutic effect (2, 4, 5, 48).

A therapeutic effect of the intervention in glycemic control could be related to the ASCs, VitD or more probably to the combination of both acting in multiple pathways to arrest β cell destruction. The inverse correlation between VitD levels and both insulin requirements and HbA1C seen in group 1 suggests a role of VitD in the process (26–28). However, most results with vitamin D supplementation from previous clinical trials are inconsistent and do not indicate a significant effect on glycemic indices in patients with recent-onset T1D (29, 31). Interestingly, Gabbay et al. have shown lower CP decline in patients with recent-onset T1D that received vitamin D supplementation (30).

Although it is not possible to determine if a potential beneficial effect of the combined intervention was caused by immune modulation or by the differentiation of ASCs in β cells, the higher frequency of CD8+FoxP3+ T cells in those that underwent the intervention suggests an increase in T cell regulatory response. Immunoregulatory CD8+ T cells have been reported before (49) and were also associated with clinical response in a T1D trial with humanized Fc-mutated anti-CD3 monoclonal antibody hOKT3 (50). However, as CD127 staining or other markers for cell characterization were not performed, we cannot exclude that the higher FOXP3 expression represents solely T cell activation (51, 52).

Both CD4+FoxP3^**+**^ and CD8+FoxP3^**+**^ cells were inversely correlated not only to HbA1C but also to various metrics of glucose variability, which suggests that a T cell regulatory response in early onset T1D might reduce glucose excursions, but might also be related to T cell activation. (2, 6–8, 50–52).

This study had some limitations. Firstly, the small sample size could explain the lack of statistical significance observed in some parameters. However, this was a pilot study with the priority to establish the safety of ASCs and the feasibility of the trial. Other limitations were the lower baseline CP and age in the control group, when compared to the intervention group. Nevertheless, we compared the percentage of changes in CP, insulin requirements and A1c variation as well as longitudinal changes in these parameters within each group. An additional limitation was the absence of a group treated solely with vitamin D and insulin, to determine if the positive results were related to ASCs, vitamin D or both. Moreover, a longer follow-up is still necessary to determine the long-term safety and efficacy of this intervention. The use of Glucerna® instead of higher glycemic index carbohydrate preparations for CP evaluation might also be a limitation as this might have slightly underestimated pancreatic residual secretion but the same test was performed for all subjects with the carbohydrate content recommended for mixed meal test.

In summary, this was the first study to test allogenic ASCs infusion + VitD without immunosuppression in patients with recent onset T1D. After a 3 month follow-up, patients that underwent intervention showed stability of CP, better glucose control and lower insulin requirement than patients that received only standard treatment, but the favorable outcome may be related to random baseline differences between groups. A larger sample and a longer follow-up period are necessary to further investigate the safety of the treatment and to determine the efficacy of ASCs infusion combined to VitD supplementation for recent-onset T1D. However, this pilot study is an important early assessment of ASCs and vitamin D supplementation as a potential combined therapy for T1D.

## Data Availability Statement

All datasets generated for this study are included in the article/supplementary material.

## Ethics Statement

This clinical trial has been registered at ClinicalTrial.gov (NCT03920397) and approved by the Ethics Research Board of the University Hospital Clementino Fraga Filho (HUCFF) from the Federal University of Rio de Janeiro (UFRJ). The patients/participants provided their written informed consent to participate in this study.

## Author Contributions

DA, MP, CC-D-S, and DS researched data. JD, KS, and LB wrote the manuscript and researched data. MR, CR, and LZ reviewed/edited the manuscript and contributed with the discussion. RL and JM contributed with statistical analysis supervision. CC, MG, SD, JO, and AM contributed to the discussion and reviewed the manuscript. PB, AS, and DD processed the ASC cells.

## Conflict of Interest

The authors declare that the research was conducted in the absence of any commercial or financial relationships that could be construed as a potential conflict of interest.
