# Supplementary material for: Allogenic Adipose Tissue-Derived Stromal/Stem Cells and Vitamin D Supplementation in Patients With Recent-Onset Type 1 Diabetes Mellitus: A 3-Month Follow-Up Pilot Study
Source: Front Immunol. 2020 Jun 2;11:993. doi: 10.3389/fimmu.2020.00993 (PMC7280537; doi:10.3389/fimmu.2020.00993)
Supplement: Supplementary file 1 [file Data_Sheet_1.PDF]

Marker	CD 105	CD 73	CD 90	CD 29	CD 166	CD 44	CD 36	CD 14	CD 34	CD 45	CD 19	HLA-DR	CD 31	CD 106
mean	93.47	96.20	99.77	98.83	94.75	89.76	19.83	1.57	0.52	0.84	0.64	0.68	0.59	4.27
SD	4.27	2.62	0.16	0.94	2.64	3.15	11.19	0.77	0.47	0.74	0.42	0.50	0.31	3.93

Legend: FITC-labeled CD14 (BD#555397), CD45 (BD#555482), CD19 (BD#555412), CD44 (BD#555478); PE-labeled CD73 (BD#550257), CD90 (BD#555596), CD166 (BD#559263), PerCP-labeled HLA-DR (BD#551375); APC-labeled CD34 (BD#555824), CD105 (BD#562408), CD29 (BD#559883) all purchased from BD (Pharmingen). At least 100.000 events were acquired on a BD FACSCalibur™ flow cytometer (BD Biosciences), and data were analyzed using FlowJo 10 (TreeStar) software11A.
