# Supplementary material for: Allogenic Adipose Tissue-Derived Stromal/Stem Cells and Vitamin D Supplementation in Patients With Recent-Onset Type 1 Diabetes Mellitus: A 3-Month Follow-Up Pilot Study
Source: Front Immunol. 2020 Jun 2;11:993. doi: 10.3389/fimmu.2020.00993 (PMC7280537; doi:10.3389/fimmu.2020.00993)
Supplement: Supplementary file 2 [file Data_Sheet_2.PDF]

Sensor Data (mg/dl)

Group 1 #A

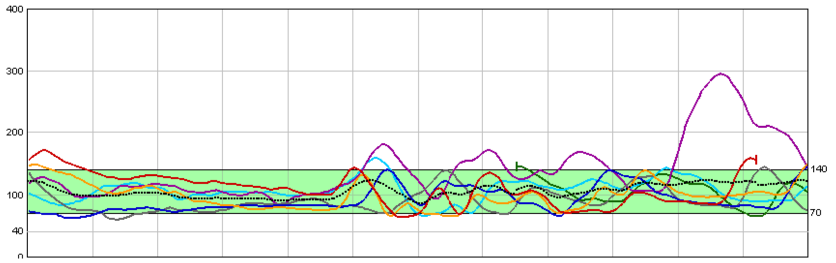

Group 1 #B

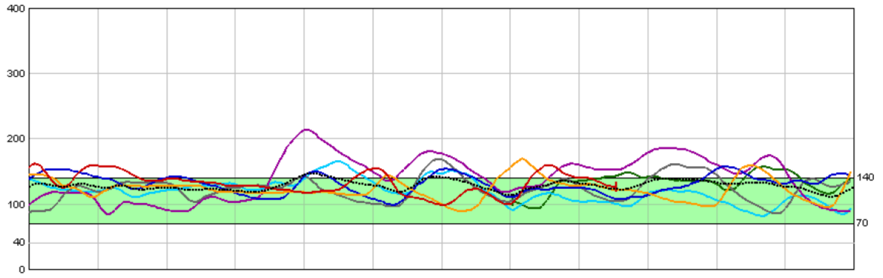

Group 1#C

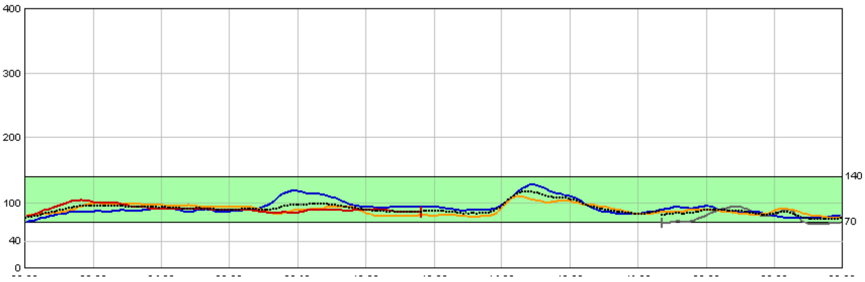

Group 1 #D

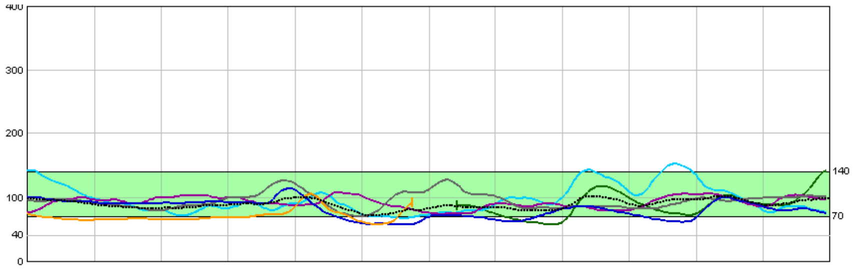

Group 1 #E

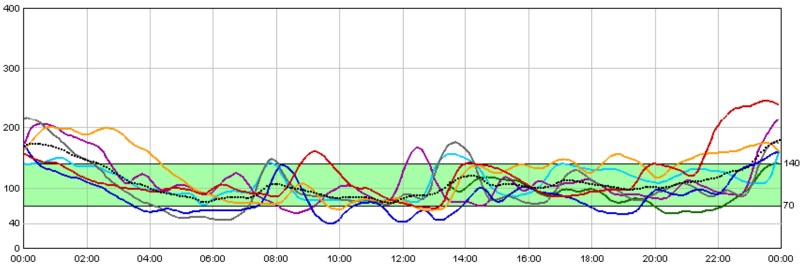

Group 1 #F

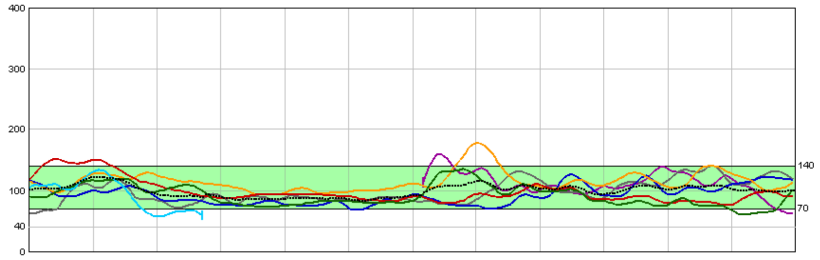

Group 1 #G

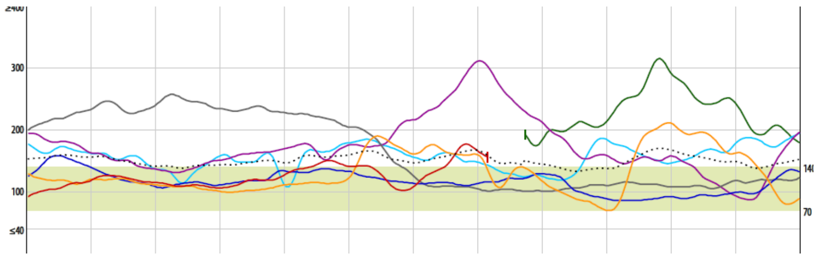

Group 2 #H

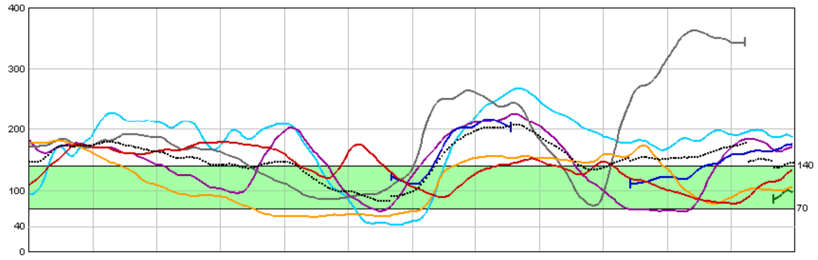

Group 2 #I

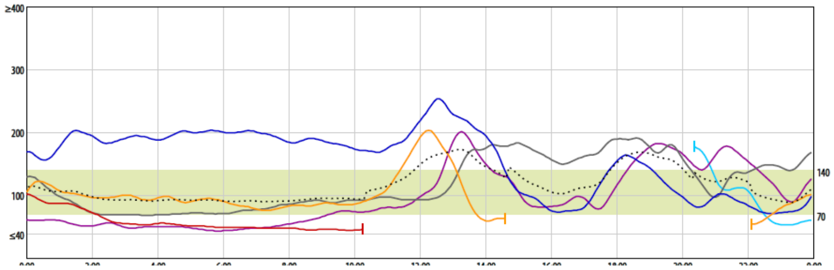

Supplementary figure : Glucose profiles achieved by retrospective continuous glucose monitoring systems. Each panel represents the glucose profiles of single individuals, of consecutive days (represented by different lines). In panel A – G, glucose profiles of group 1 three months after ASCs infusion + VitD oral supplementation are shown. In panel H-I, glucose profiles of group 2 are shown.
