# Supplementary material for: Allogenic Adipose Tissue-Derived Stromal/Stem Cells and Vitamin D Supplementation in Patients With Recent-Onset Type 1 Diabetes Mellitus: A 3-Month Follow-Up Pilot Study
Source: Front Immunol. 2020 Jun 2;11:993. doi: 10.3389/fimmu.2020.00993 (PMC7280537; doi:10.3389/fimmu.2020.00993)
Supplement: Supplementary file 3 [file Data_Sheet_3.PDF]

Number	Patient	Gender	Age	Ethnicity	BMI	Months T1D diagnosis	ASCs number	Insulin dose(IU/ Kg) T0
1	JL	1	26	1	26,06	4	78	0,84
2	MA	1	35	2	25,91	4	74	0,17
3	MV	1	28	1	23,38	2	65	0,21
4	DC	2	34	1	23,56	2	73	0,07
5	RM	2	23	2	20,76	1,7	60	0,15
6	NO	2	16	1	20,96	3,5	55	0,47
7	RM	2	28	1	23,71	2	69	0,25
8	LB	1	24	1	24,2	4	66	0,3
9	WS	1	16	1	18,25	2		0,92
10	BG	2	20	2	23,71	3		0,92
11	PM	1	18	2	18,2	3		0,6
12	EC	2	25	1	20,6	3		0,2
13	JM	1	24	1	19,3	4		0,5

Missing data due to dosage or device problems

Insulin dose (IU/Kg) T1	Insulin dose(IU/Kg) T3	A1C TO	A1C T1	A1C T3	GADA T0	GADA T1	GADA T3	IA2A TO	IA2A T1
0,64	0,47	9,9	7,6	6,2	60,88	107,31	62,07	13	13,98
0,15	0,18	8	7,5	6,6	92,11	54,41	53,11	10,48	10,48
0,18	0,17	6,3	5,6	5,5	344,51	54,41	290,38	10,48	10,48
0,07	0,11	7,5	6,9	6,2	285,71	306,49	294,93	10,48	10,48
0,15	0,12	7,9	6,3	6,4	279,87	290,25	286,88	596,68	589,79
0,47	0,49	6,9	5,8	5,7	289,22	248,96	258,7	10,48	10,48
0,2	0,25	7,6	6,6	6,9	241,29	332,87	335,95	596,68	589,79
0,18	0	7,4	7,2	8,3	17,27	4000	4000	18	17,97
0,82	0,92	10,6	6,9	7,8	7,01	7,14	7,53	10,48	10,48
0,69	0,64	6,8	6,8	6,9	340,49	358,18	407,5	18,66	19,35
0,6	0,7	6,9	7,4	7,2	274,45	279,51	276,14	4000	4000
0,2	0,2	7,7	7,1	8,2	127,76	44,45	500	18,38	18,32
0,5	0,6	10,1	7,4	7,3	160,95	35,08	34,25	46,2	40,15

IA2A T3	TSH T0	TSH T1	TSH T3	Vitamin D T0	Vitamin D T1	Vitamin D T3	FOXP3 + CD4+ (%) TO	FOXP3 + CD4+ (%) T1	FOXP3 + CD4+ (%) T3
13,93	5,64	10,5	9,66	40,3	39,3	50,6	13,3	19,9	13,8
10,48	2,75	2,35	2,13	16,4	35,7	36,5	20,8	18,9	18,8
10,48	6,78	4,42	4,16	12	27	42,3	9,5	11,8	19,5
10,48	1,35	1,6	1,85	36	42,7	54,3	6,6	25,1	14
617,37	1	0,818	0,986	37,5	47,5	59,6	2,6	12,9	15,3
7,03	0,82	1,25	0,82	40,5	37,9	43,9	19,5	25,3	30
617,37	8,6	5	5,61	48,7	40,5	41,5	1,8	18,8	4,3
18,45	1,16	0,8	1,01	44,8	68	56,55	7,7	7,7	1,3
10,48	5,59	5,78	5,49	24,3	29,7	23,9	25,4	13,9	15,4
18,35	4,63	5,91	6,37	10,6	16	29,9	5,5	10,5	5,8
4000	1,18	1,28	0,896	19,3	23,5	20,4	8	14,7	18,1
18,03	1,08	1,15	0,759	26,6	32,2	24,4	17,4		11,3
32,01	6,333	3,28	3,739	27,9	37,7	32,88	20,1	7,8	3,2

THIS IS NOT INTENDED FOR PUBLICATION. THIS IS SUPI

FOXP3+ CD8+ (%) TO	FOXP3+ CD8+ (%) T1	FOXP3+ CD8+ (%) T3	CP AUC T0	CP AUC T1	CP AUC T3	Mean T0	Mean T3	SD T0	SD T3
21,9	13,1	4,5	104,85	63,6	157,05	7,2468	6,2875	2,0807	1,8474
15,3	16,7	11,4	148,95	162,6	149,7	6,4931	7,1489	0,7607	1,3522
13,6	27,5	17,1	340,5	470,7	388,95	4,9596	4,9757	0,9023	0,5396
18,9	24	16	328,5	317,7	291,9	7,0785	5,1966	1,6108	0,9017
10,5	6,1	5,7	178,95	158,85	99,3	5,9945	5,9225	1,6914	1,7933
28,8	15,4	27,4	318,75	354,3	251,1	6,592	5,5579	1,7763	0,9971
0	16,5	3,1	233,7	270	243,45	7,1898	9,92	1,5236	2,5641
1,8	7,5	0,6	153	204	108,15	9,9924	9,3124	3,012	3,3778
10,2	3,6	21,4	86,25	140,7	173,7	7,815	6,9454	2,8309	2,3624
4,6	8,1		90,3	127,95	78,75	8,2248		1,9657	
5,4	5,6	8,4	102,75	118,65	106,2	6,0815		0,7266	
2,8		14,9	160,2	147,9	123,15				
4,6	6,2	0,3	113,25	101,55	48,45				

PLEMENTARY DATA SENT TO REVIEWERS AND EDITOR

M-VALUE T0	M-VALUE T3	MAGE T0	MAGE T3	J-INDEX T0	J-INDEX T3	GRADE T0	GRADE T3	LBGI T0	LBGI T3
1,3219	0,2	1676,4	1737	28,35	21,9737	0,2319	0,2142	7,1246	2,4233
0,0533	0,8588	826,8	1431	18,3345	23,4447	0,0701	2043,682	0,7662	0,6688
-4,07	-2,9095	1093,8	0	11,2405	9,8676	2,0483	1,9161	3,0539	2,4093
0,1165	-3,1521	1470	1273,8	24,748	12,0726	0,2345	1,4673	1,7087	2,6217
-2,2972	-3,6818	1594,8	1827,6	19,1959	19,4379	0,418	0,5296	3,1312	3,8263
-1,0177	-2,1184	1494,6	708	22,7194	13,9246	0,0843	0,8169	2,7261	2,19759
0,9594	10,3984	1546,8	2136	24,6264	50,5078	0,0261	1,6961	1,0897	0,1949
12,5943	8,5538	8,7052	10,1114	55,4922	53,03	1,7887	3,2601	2,4429	6,2683
6,7434	9,1914	8,4771	6,7027	37,9934	29,0002	0,9791	0,9928	5,3141	6,1707
3,2469		6,4018		33,7495		0,4276		1,4719	
0,8407		2,9018		16,433		0,4276		1,5182	

HGBI T0	HGBI T3	MODD TOxT3	CONGA1 T0	CONGA1 T3	%Time in range T0	%Time in range T3
12,4851	3,663	2,3683	1,1847	1,1847	86	92
1,1113	2,4627	1,8009	0,5547	0,5547	99	97
1,4796	0,0464	1,2425	1,0265	2,2417	95	97
3,2261	0,7262	1,9753	2,2417	2,2417	91	96
2,3141	3,0275	2,2284	0,9382	0,9382	91	87
2,7529	0,5518	2,0052	1,0921	1,0921	96	97
2,5174	9,8337	2,8711	0,3733	0,3733	91	60
10,5739	11,0329	3,2971	3,5018	2,3348		
6,0544	5,8205	3,2787	2,1209	1,8272	57	51
4,79			2,2528		79	64
0,7776			1,3711		88	
					100	
